# Optimizing Chest X-ray Referral Using Clinical Metadata: Pulmonary Disease Patterns and Diagnostic Access in Southwestern Uganda

**DOI:** 10.7759/cureus.115057

**Published:** 2026-08-23

**Authors:** Johnes Obungoloch, Julius Tumusiime, Gershom Buri, Martin Mukama, Jacob Nkwanga, Chrispus Mbusa, Fred Kaggwa, Shallot N Murungi, William Wasswa

**Affiliations:** 1 Biomedical Engineering, Mbarara University of Science and Technology, Mbarara, UGA; 2 Computer Science, Mbarara University of Science and Technology, Mbarara, UGA; 3 Centre for Affordable Medical Technologies (CAMTech), Mbarara University of Science and Technology, Mbarara, UGA; 4 Internal Medicine, Kabale University, Kabale, UGA; 5 Administration, Mbarara University Data Science Research Hub, Mbarara, UGA; 6 Faculty of Computing and Informatics, Mbarara University of Science and Technology, Mbarara, UGA; 7 Biomedical Sciences and Engineering, Mbarara University of Science and Technology, Mbarara, UGA

**Keywords:** chest x-ray, clinical metadata, diagnostic imaging, healthcare access, predictive modelling, pulmonary disease, risk stratification, southwestern uganda

## Abstract

Background: Chest X-ray (CXR) imaging is important for diagnosing pulmonary and cardiothoracic conditions, but timely access remains limited in many low- and middle-income countries. This study characterized radiographic abnormalities, examined associated clinical factors, evaluated exploratory clinical metadata-based prediction models, and assessed barriers to CXR utilization in southwestern Uganda.

Methods: This facility-based observational pilot study included 422 adults undergoing chest radiography for suspected pulmonary or cardiothoracic disease at two healthcare facilities. Prospectively collected demographic, clinical, environmental, and healthcare-access data were linked to routine radiographer reports. Radiographer-reported pneumonia, pleural effusion, and cardiomegaly were summarized descriptively. Associated factors were examined using multivariable logistic regression with complete-case analysis. Exploratory model discrimination was assessed using receiver operating characteristic analysis, while post hoc Stage 1 simulations examined trade-offs between imaging-referral volume and case detection.

Results: Complete radiographic-outcome classifications were available for 403 participants. Pneumonia was the most frequently reported abnormality (18.9%), followed by pleural effusion (9.7%) and cardiomegaly (5.5%). Increasing age was independently associated with pneumonia (adjusted odds ratio (aOR) 1.318 per 10-year increase; 95% confidence interval (CI) 1.149-1.523) and cardiomegaly (aOR 1.756 per 10-year increase; 95% CI 1.383-2.240), but not pleural effusion. Higher body mass index was associated with lower odds of pleural effusion and slightly higher odds of cardiomegaly. The exploratory models showed apparent in-sample discrimination for cardiomegaly (AUC 0.881; 95% CI 0.807-0.963), pneumonia (AUC 0.787; 95% CI 0.728-0.849), pleural effusion (AUC 0.699; 95% CI 0.601-0.788), and any reported abnormality (AUC 0.790; 95% CI 0.742-0.836). Effective access declined after accounting for personnel availability, affordability, and willingness to undergo imaging; only 17% of participants reported being both willing and able to complete the diagnostic pathway. The Stage 1 simulations illustrated trade-offs between referral volume and case detection under selected thresholds.

Conclusions: Routinely obtainable clinical metadata may contain useful information for preliminary pre-imaging risk stratification. However, the reported AUCs represent apparent discrimination within the model-development sample, and the referral strategies were neither prospectively implemented nor clinically validated. The findings are exploratory and hypothesis-generating. Independent radiologist verification, validation of the report-classification procedure, model calibration, internal and external validation, and prospective workflow evaluation are required before the proposed approach can support patient-level referral decisions.

## Introduction

Chest radiography remains one of the most widely used and accessible diagnostic imaging examinations for detecting and monitoring pulmonary and cardiothoracic conditions, including pneumonia, tuberculosis, lung cancer, pleural disease, and cardiovascular abnormalities [1,2]. In sub-Saharan Africa, where respiratory and cardiothoracic diseases continue to impose a substantial burden on healthcare systems, chest radiography plays an important role in clinical decision-making [3,4]. However, access to imaging and timely expert interpretation remains limited in many low- and middle-income countries (LMICs), including Uganda. Shortages of radiologists mean that chest radiographs are often interpreted by other healthcare professionals with varying levels of radiological expertise, which may contribute to delayed or inaccurate diagnosis [3].

These limitations affect both the interpretation of completed examinations and the decisions that determine which patients receive imaging. Although chest radiograph interpretation conventionally relies on trained radiologists, their scarcity in resource-limited settings frequently requires general clinicians to interpret radiographic findings despite limited specialized training [4,5]. At the same time, constrained equipment, staffing, affordability, and referral systems may prevent clinically eligible patients from accessing chest radiography. These challenges are compounded by fragmented health information systems in which imaging findings, clinical history, vital signs, and other patient information are recorded separately, limiting comprehensive assessment and informed decision-making.

Recent advances in artificial intelligence (AI) and machine learning have demonstrated potential to support chest X-ray (CXR) interpretation, improve diagnostic accuracy, and strengthen clinical workflows [6-8]. However, the development and deployment of reliable models depend on large, high-quality, and representative datasets. Most existing models have been developed using data from high-income countries and may not generalize adequately to African populations because of differences in disease epidemiology, patient characteristics, environmental exposures, healthcare access, and imaging practices [9,10]. The limited availability of linked clinical and imaging datasets from African healthcare settings therefore remains an important barrier to developing locally relevant and clinically useful decision-support tools.

Approaches that use routinely collected clinical information may help address the decision point before imaging is performed. Patient demographics, presenting symptoms, vital signs, environmental and behavioral exposures, and comorbidities may support early risk stratification and help prioritize referrals when imaging capacity is constrained [11,12]. When subsequently linked with chest-radiographic outcomes, these data can also be used to examine disease patterns and develop contextually relevant prediction models [13]. Importantly, the Stage 1 referral strategies evaluated in this study represented pre-imaging triage: referral decisions were derived from analysis of clinical presentation and routinely collected patient parameters only. Radiographer-reported imaging findings were not used to determine these simulated referrals.

To enable these analyses, we established a multimodal database linking CXR examinations with structured clinical metadata collected from patients receiving routine care in southwestern Uganda. This dataset provided a common framework for describing chest radiographic abnormalities, examining their clinical correlates, evaluating metadata-based prediction, assessing access to imaging, and exploring how limited chest-radiography capacity might be allocated through clinically informed referral strategies.

The primary objectives were to characterize the prevalence of major chest-radiographic abnormalities and identify clinical factors independently associated with pneumonia, pleural effusion, and cardiomegaly. The secondary objectives were to evaluate the internally validated discriminatory performance of clinical metadata-based prediction models, assess barriers to chest radiography access, and simulate Stage 1 referral strategies under different imaging-capacity assumptions. By integrating epidemiological analysis, prediction modelling, and health-system evaluation, the study sought to determine how routinely collected clinical information could support more efficient, equitable, and resource-aware diagnostic pathways in a low-resource healthcare setting.

## Materials and methods

Study design and setting

This observational pilot study, conducted in a facility-based setting, utilized prospectively collected clinical and radiographic data from consenting adults who underwent chest radiography as part of routine care between March 25, 2024, and May 31, 2025. Data collection adhered to a predefined study protocol, and the analyses presented here were performed following the completion of enrolment. The individual patient encounter served as the unit of analysis, with each record comprising a clinical assessment linked to a corresponding chest radiograph.

Participants were recruited from Divine Mercy Hospital and Mbarara Regional Referral Hospital in southwestern Uganda. All study-related chest radiographs were obtained at Divine Mercy Hospital using a digital radiography system. Participants identified at Mbarara Regional Referral Hospital were referred to Divine Mercy Hospital due to the inconsistent availability of comparable digital imaging at the referral hospital during the study period. Consequently, recruitment involved two levels of healthcare delivery, while image acquisition was centralized at one institution.

Participant recruitment

Potential participants were systematically approached from among patients who had been clinically referred for chest radiography due to suspected pulmonary or cardiothoracic disease. Consequently, the study did not involve an unselected community or primary-care screening population. Eligibility criteria included adults aged 18 years or older who could provide written informed consent and undergo erect chest radiography. Exclusion criteria encompassed patients who were critically ill or unable to undergo chest radiography in the erect position.

**Figure 1:**
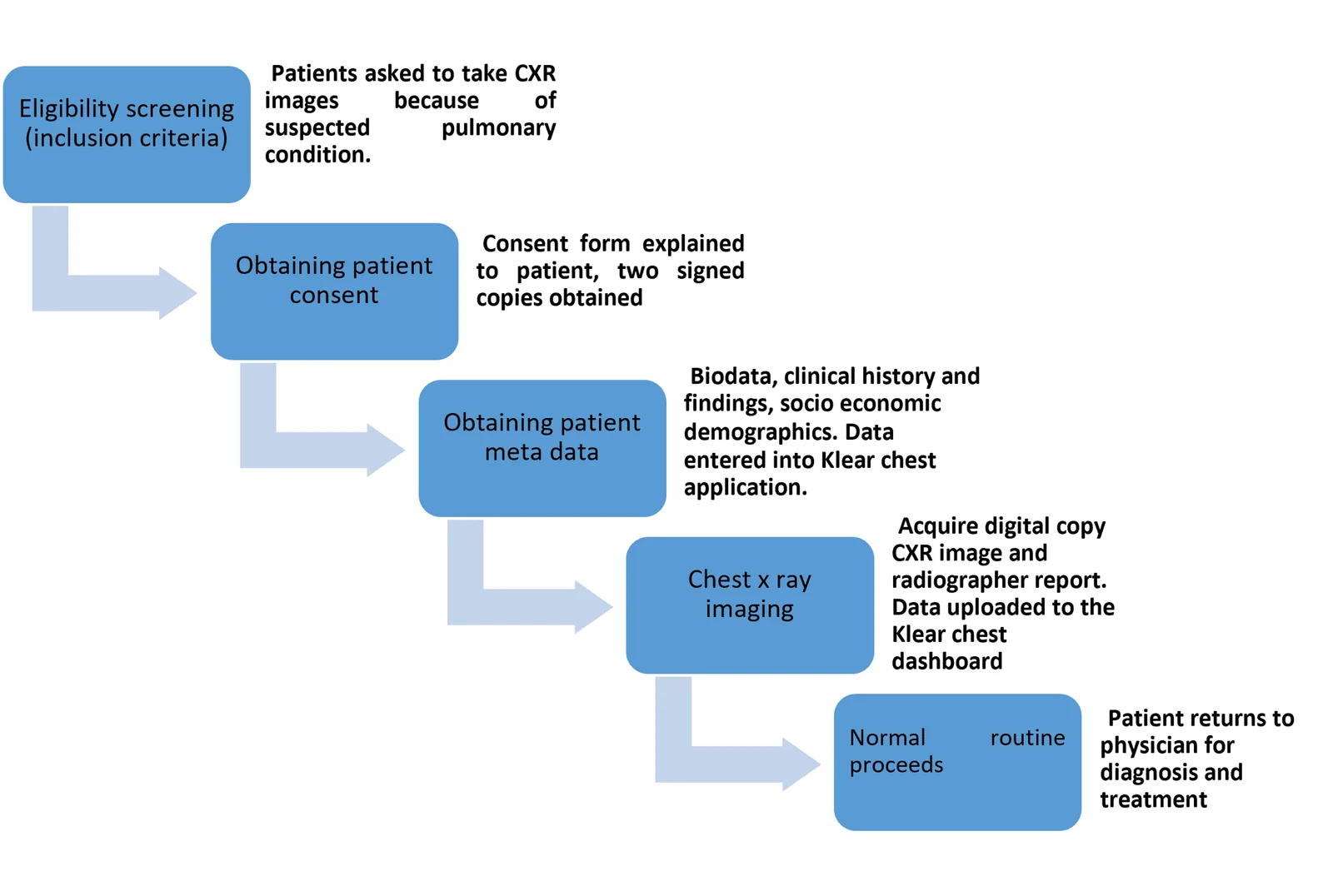
Patient handling and data acquisition procedure CXR: Chest X-ray

During the recruitment period, 475 individuals were approached. Of these, 47 declined participation, resulting in 428 individuals being assessed for eligibility. Two individuals were excluded for being under 18 years of age, and four were excluded due to critical illness. Ultimately, 422 participants provided written informed consent and were enrolled in the study. Following consent, participants underwent the standard clinical workflow, which included medical history documentation, physical examination, chest radiography, and routine radiographic reporting. The participant flow and data acquisition process are depicted in Figure 1.

Data collection and management

Clinical and radiographic data were collected prospectively through standardized study procedures. While clinical variables comprised information typically obtained during patient assessments, they were entered into the research database only after enrolment and the acquisition of written informed consent. The structured metadata encompassed sociodemographic characteristics, socioeconomic indicators, behavioral factors, environmental exposures, presenting symptoms, vital signs, comorbidities, and variables related to access to and use of imaging services. Clinical information was electronically entered using the custom-developed Klear Chest data-collection platform. CXR images and their corresponding narrative reports were uploaded via a secure web-based dashboard to servers managed by the Data Management and Analysis Core of the Mbarara University Data Science Research Hub. Each participant provided one clinical record and one associated chest radiograph. Records were subsequently reviewed, digitized as necessary, and integrated into a structured analytic dataset.

Radiographic outcomes and report classification

The primary outcomes focused on identifying the presence or absence of three significant chest radiographic abnormalities: pneumonia, pleural effusion, and cardiomegaly. These outcomes were extracted from routine chest radiograph reports, which were generated by degree-level radiographers or medical imaging experts with a minimum of five years of experience as part of their service delivery. Due to the lack of an independent expert radiologist consensus interpretation, these outcomes are consistently referred to as radiographer-reported abnormalities rather than definitive diagnoses. A deterministic, rule-based text classification procedure was employed to convert narrative reports into binary outcome labels. For each outcome, the reports were scrutinized for predefined diagnostic terms and their accepted synonyms, as detailed in Table 1. Explicit negations, such as 'no,' 'without,' 'absent,' 'free of,' and 'ruled out,' were categorized as indicating the absence of the corresponding finding. This procedure aimed to standardize existing narrative reports and did not involve a trained natural language processing or machine learning model. Statements indicating uncertainty or a differential diagnosis, including terms like 'query,' 'cannot exclude,' 'suggestive of,' and 'rule out,' were classified as possible. After applying the terminology, negation, and uncertainty rules, each target outcome was coded as either present or absent. The rule set was not formally validated against independent manual classification or an expert reference standard. 

**Table 1:** Rule-based classification table for target abnormality

Target abnormality	Accepted terms and synonyms	Negation terms	Uncertainty terms	Classification decision
Pneumonia	Pneumonia; bronchopneumonia; pneumonic consolidation; recognized spelling/misspelling variants	No, without, absent, free of, ruled out, other complication	rule out, r/o, suggestive of, suggest, likely, to be ruled/r/o	Present/possible/absent
Pleural effusion	Pleural effusion; hydrothorax; recognized spelling/misspelling variants (e.g. plueral)	No, without, absent, free of, ruled out, other complication	rule out, r/o, suggestive of, suggest, likely, to be ruled/r/o	Present/possible/absent
Cardiomegaly	Cardiomegaly; recognized synonym/misspelling variants	No, without, absent, free of, ruled out, other complication	rule out, r/o, suggestive of, suggest, likely, to be ruled/r/o	Present/possible/absent

Data processing

Prior to analysis, variable names, formats, and category labels were standardized across data sources. Sparse categorical levels were consolidated only when clinically and conceptually justified. Physiological measurements deemed clinically implausible were treated as missing, based on the prespecified ranges detailed in Table 2. 

**Table 2:** Plausible ranges of vital clinical parameters for inclusion or exclusion in the analysis

Variable	Minimum accepted value	Maximum accepted value	Treatment of out-of-range values.
Systolic blood pressure	90	140	Missing
Diastolic blood pressure	50	80	Missing
Oxygen saturation	60	100	Missing
Respiratory rate	10	23	Missing
Temperature	36	37	Missing
BMI	18	40	Missing

The extent of missing data was evaluated for each variable. No statistical imputation was conducted. Descriptive analyses utilized all available observations, with variable-specific denominators reported when data were incomplete. Multivariable analyses employed complete cases for the variables included in each outcome-specific model, resulting in differing analytical sample sizes across models. Healthcare-access and diagnostic-pathway variables were analyzed separately from disease-specific explanatory models.

Statistical analysis

Participant characteristics, clinical presentations, radiographer-reported abnormalities, comorbidities, environmental exposures, and healthcare-access indicators were summarized descriptively. Categorical variables were presented as frequencies and percentages, utilizing the available denominator. Associations between participant characteristics and conditions such as pneumonia, pleural effusion, and cardiomegaly were analyzed using separate multivariable logistic regression models. Results are expressed as adjusted odds ratios with 95% confidence intervals and p-values. For continuous predictors, the effect unit was the original measurement unit unless otherwise specified in the relevant results table.

The candidate covariate set included age, sex, body mass index, presenting symptoms, smoking status, alcohol-use score, hypertension, education, income, household fuel type, cooking location, and other recorded clinical or exposure variables. The final pneumonia model incorporated age, sex, smoking status, cooking location, household fuel type, education, and income score. The pleural effusion model included age, sex, body mass index, hypertension, and education. The cardiomegaly model comprised age, hypertension, body mass index, and AUDIT-C score. The pneumonia, pleural effusion, and cardiomegaly models included 396 participants (74 events), 395 participants (39 events), and 398 participants (21 events), respectively.

The regressions were exploratory in nature. Formal assessments of multicollinearity, linearity of continuous predictors in the logit, interaction effects, and influential observations were not conducted. Exploratory models were developed separately for pneumonia, pleural effusion, cardiomegaly, and the presence of any reported radiographic abnormality. Predictors were limited to demographic and clinical information available prior to the interpretation of the chest radiograph. Radiographic findings and radiographer reports were solely used to define outcome labels and were not included as model predictors.

Discrimination was quantified using the area under the receiver operating characteristic curve (AUCs). Predictions used to estimate the reported AUCs were generated from the same dataset used for model development; thus, the estimates represent apparent in-sample discrimination. Cross-validation, bootstrap optimism correction, evaluation in a held-out test set, calibration assessment, and external validation were not performed. The models were exploratory and not intended to serve as validated clinical prediction tools. Healthcare-access variables were analyzed descriptively to examine sequential barriers along the chest-radiography pathway, including geographical availability of services, personnel availability, affordability, referral requirements, and willingness or ability to undergo imaging.

Ethical considerations

Ethical approval was obtained from the Mbarara University of Science and Technology Research Ethics Committee (MUST-REC; Registration No. MUST-2024-1343) and the study was subsequently approved by the Uganda National Council for Science and Technology (UNCST; Registration No. SS2718ES). All participants provided written informed consent before enrolment. Chest radiographs and associated clinical metadata were de-identified before analysis to protect participant confidentiality and were managed in accordance with the Uganda Personal Data Protection and Privacy Act (2019).

## Results

Participant characteristics

A total of 422 eligible and consenting adults undergoing chest radiography for suspected pulmonary or cardiothoracic conditions were enrolled in the study. The study population encompassed a diverse range of demographic and socioeconomic backgrounds. The mean age of participants was 48.2 ± 21.2 years, with individuals aged 50 years and above constituting the largest age group (n = 215, 51%). The majority of participants were economically inactive (n = 145, 34%), while 134 (32%) were engaged in agriculture or informal occupations, 53 (13%) had formal employment, and 20 (4.7%) were classified under other occupations. Educational attainment varied across the study population, with secondary education being the most common level achieved (n = 220, 53%), followed by tertiary education (n = 106, 25%), primary education (n = 54, 13%), and no formal education (n = 39, 9.3%). Exposure to biomass fuels and other environmental risk factors was prevalent among all participants, reflecting the predominantly rural and peri-urban settings from which the study population was drawn. Body mass index and other physiological measurements were recorded as indicators of general health status and were subsequently evaluated in multivariable analyses.

Clinical presentation

**Figure 2:**
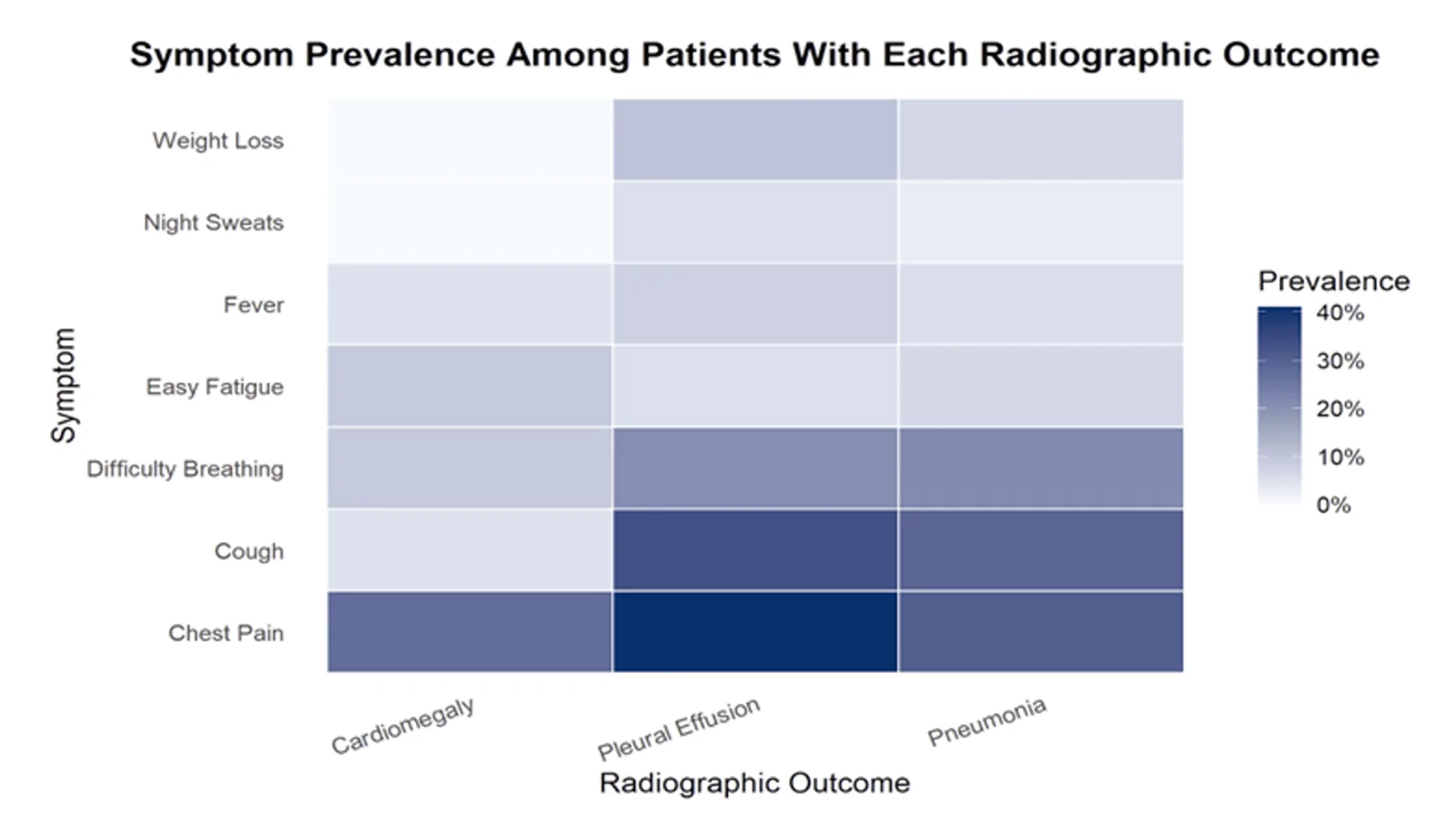
Symptom prevalence among patients

Respiratory and constitutional symptoms were frequently reported among participants. Cough, chest pain, and difficulty breathing were the most common presenting symptoms and were observed across all major radiographic conditions. Fever, weight loss, night sweats, and fatigue were also reported but exhibited similar prevalence patterns across disease categories. No individual symptom was unique to a specific radiographic diagnosis, indicating substantial overlap in clinical presentation among patients undergoing chest radiography (Figure 2).

Radiographic findings

Radiographic examination identified a spectrum of pulmonary and cardiothoracic abnormalities. Pneumonia was the most frequently reported condition, affecting 18.9% of participants, followed by pleural effusion (9.7%) and cardiomegaly (5.5%) (Table 3).

**Table 3:** Prevalence of the three common pulmonary conditions identified

Outcome	Number of participants	Participants omitted due to missing data	Participants considered	Positive cases	Prevalence (%)
Cardiomegaly	422	19	403	22	5.5
Pleural effusion	422	19	403	39	9.7
Pneumonia	422	19	403	76	18.9

**Figure 3:**
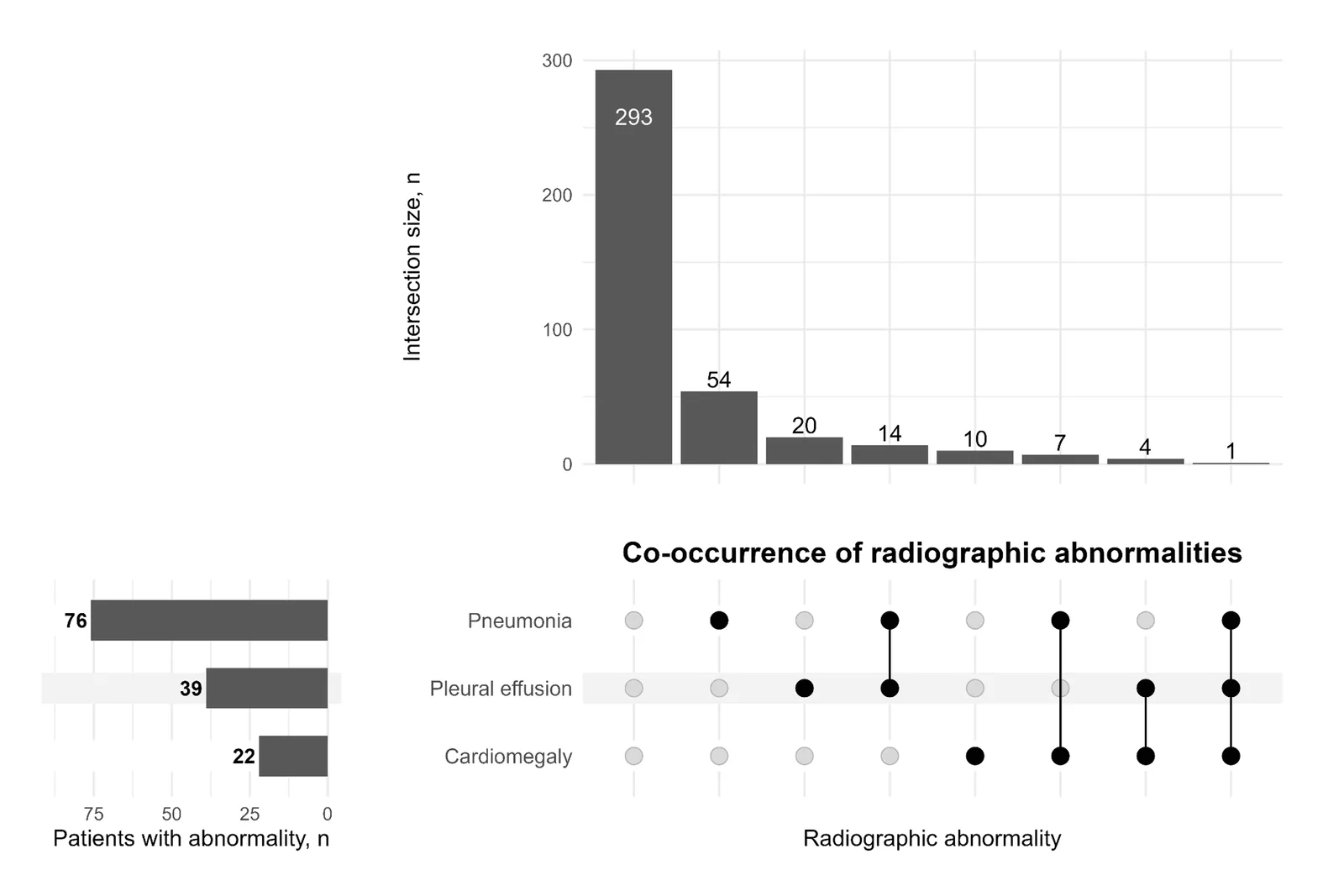
Co-occurrence of selected chest radiographic abnormalities The UpSet plot shows individual and overlapping occurrences of pneumonia, cardiomegaly, and pleural effusion. Horizontal bars indicate the total number of radiographs containing each abnormality, while vertical bars show the number of radiographs in each mutually exclusive intersection. Filled circles identify the abnormalities included in each intersection, and connected circles indicate co-occurrence within the same radiograph. Counts are based on radiographs with available classifications for all included abnormalities.

Although many participants presented with a single radiographic abnormality, multiple conditions frequently occurred in combination. The distribution of individual and overlapping abnormalities is summarized in Figure 3.

Comorbidities and risk factors

Several chronic medical conditions were documented within the study population, including hypertension and other long-term illnesses. Behavioral and environmental risk factors, such as smoking, alcohol consumption, and biomass fuel exposure, were also recorded. These characteristics were observed across the different radiographic outcomes and were subsequently evaluated in multivariable regression analyses to determine their independent associations with disease.

Factors associated with major radiographic outcomes

**Figure 4:**
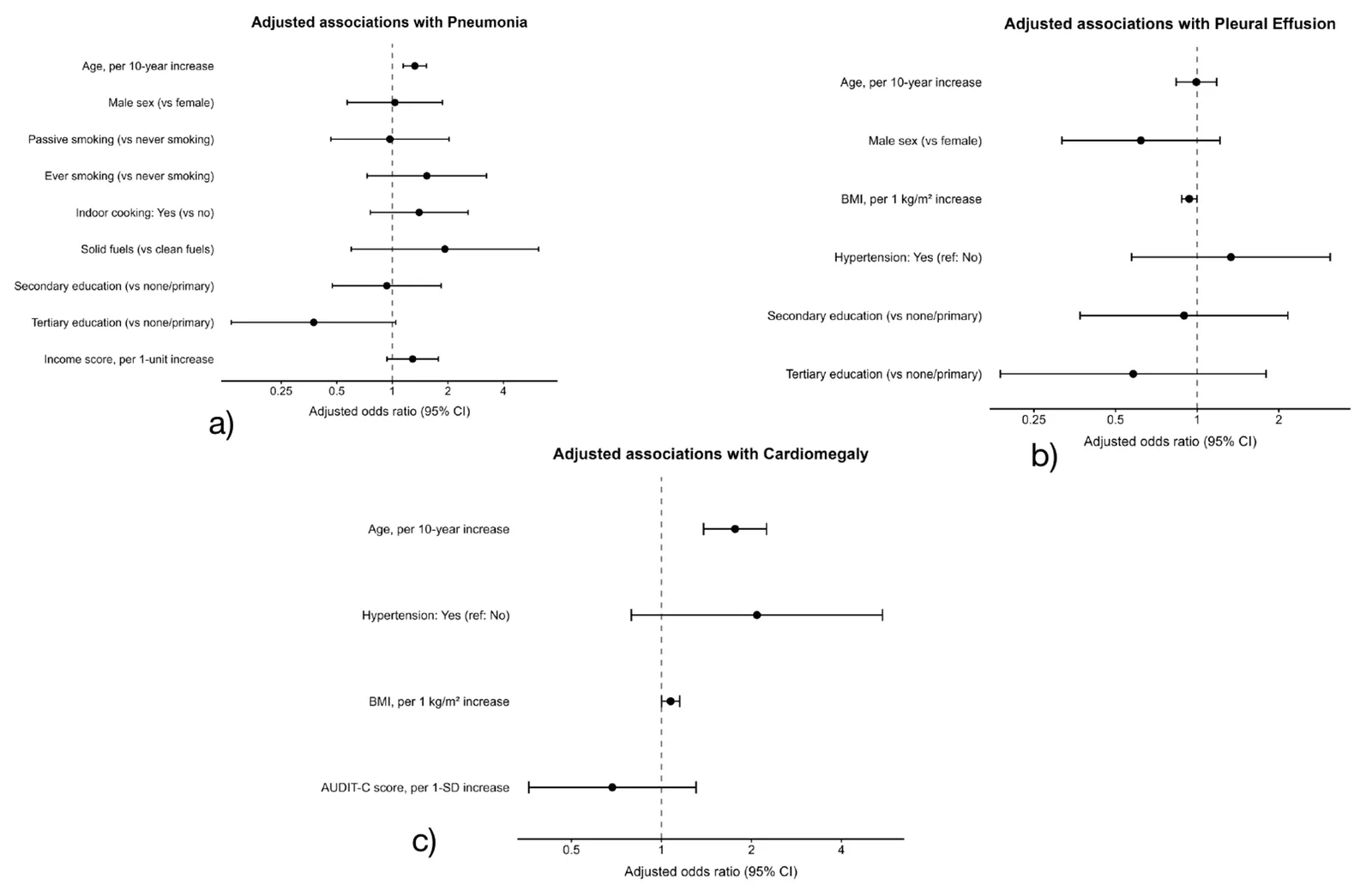
Adjusted associations between participant characteristics and selected chest radiographic abnormalities Forest plots show adjusted odds ratios (ORs) and 95% confidence intervals (CIs) for factors associated with (a) pneumonia, (b) pleural effusion, and (c) cardiomegaly. Points represent adjusted OR estimates, and horizontal lines indicate 95% CIs. The dashed vertical line marks the null value (OR = 1). Variables with ORs greater than 1 indicate higher odds of the corresponding abnormality, whereas ORs less than 1 indicate lower odds, after adjustment for other covariates included in each model.

Multivariable logistic regression analyses identified age as the most consistent predictor of major radiographic abnormalities (Figure 4). Increasing age was independently associated with higher odds of both pneumonia and cardiomegaly (Figures 4a, 4c). Body mass index showed a positive association with cardiomegaly (Figure 4c) but was inversely associated with pleural effusion (Figure 4b). Other demographic, socioeconomic, and behavioral variables, including education level, income, smoking history, hypertension, and alcohol use, were not independently associated with the major radiographic outcomes after adjustment for potential confounders.

Complete numerical regression results, including adjusted ORs, 95% CIs, p-values, units of effect, model covariates, and analytical sample sizes for pneumonia, pleural effusion, and cardiomegaly, are presented in Tables 4-6, respectively.

**Table 4:** Multivariable associations with pneumonia Analytical N = 396; events = 74; non-events = 322: Increasing age was associated with greater odds of pneumonia. For every 10-year increase in age, the adjusted odds of pneumonia increased by approximately 32% (aOR 1.318, 95% CI 1.149–1.523; p<0.001). There was some evidence of lower odds among participants with tertiary education compared with those with no or primary education, but this association did not meet the conventional 5% significance threshold (aOR 0.375, 95% CI 0.134–1.045; p=0.061).

Covariate	Unit or comparison	Adjusted OR	95% CI	p-value
Age	Per 10-year increase	1.318	1.149–1.523	<0.001
Sex	Male vs female	1.032	0.570–1.868	0.917
Smoking status	Passive vs never	0.970	0.464–2.031	0.936
Smoking status	Ever (former/current) vs never	1.537	0.730–3.236	0.258
Cooking location	Yes vs No	1.398	0.759–2.572	0.282
Household fuel	Solid fuels vs clean fuels	1.927	0.599–6.205	0.272
Education	Secondary vs no/primary	0.933	0.473–1.840	0.842
Education	Tertiary vs no/primary	0.375	0.134–1.045	0.061
Income score	Per 1-unit increase	1.288	0.935–1.775	0.122

**Table 5:** Multivariable associations with pleural effusion Analytical N = 395; events = 39; non-events = 356: Higher body mass index was independently associated with lower odds of pleural effusion (adjusted OR 0.935 per unit increase, 95% CI 0.877–0.997, p=0.042). No other covariates showed statistically significant independent associations.

Covariate	Unit or comparison	Adjusted OR	95% CI	p-value
Age	Per 10-year increase	0.990	0.833–1.183	0.940
Sex	Male vs Female	0.620	0.317–1.214	0.163
Body mass index	Per 1 kg/m² increase	0.935	0.877–0.998	0.042
Hypertension	Yes vs No	1.332	0.573–3.094	0.505
Education	Secondary vs No/Primary	0.895	0.370–2.160	0.804
Education	Tertiary vs No/Primary	0.581	0.188–1.796	0.346

**Table 6:** Multivariable associations with cardiomegaly Analytical N = 398; events = 21; non-events = 377: Older age was strongly associated with greater odds of cardiomegaly. The adjusted odds increased by approximately 76% for every 10-year increase in age (aOR 1.756, 95% CI 1.383–2.240; p<0.001). Higher body mass index was also associated with greater odds of cardiomegaly, although the magnitude of association was modest and close to the conventional threshold for statistical significance (aOR per 1 kg/m² increase 1.074, 95% CI 1.00–1.151; p=0.046). Neither hypertension nor AUDIT-C score showed statistically significant independent associations with cardiomegaly.

Covariate	Unit or comparison	Adjusted OR	95% CI	p-value
Age	Per 10-year increase	1.756	1.383–2.240	<0.001
Hypertension	Yes vs No	2.086	0.793–5.487	0.136
Body mass index	Per 1 kg/m² increase	1.074	1.001–1.151	0.046
AUDIT-C score	Per 1-SD increase	0.685	0.360–1.306	0.251

Predictive model performance

**Figure 5:**
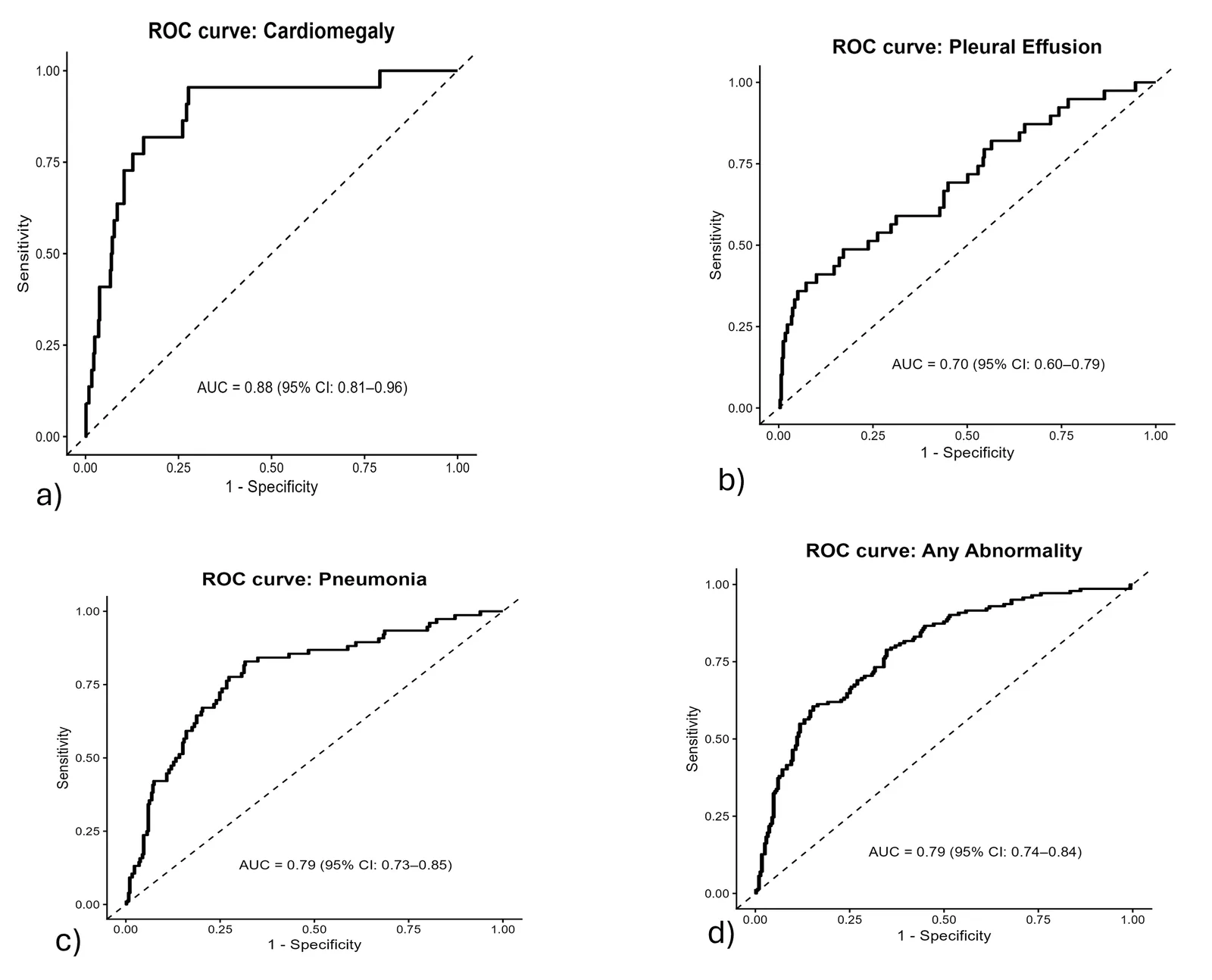
Predictive model performance a) The predictive model demonstrated excellent discrimination for cardiomegaly (AUC 0.881, 95% CI 0.807–0.963). Age was the strongest predictor of cardiomegaly, followed by systolic blood pressure and oxygen saturation. All other variables, including symptoms, contributed negligibly. b) Moderate discrimination for pleural effusion (AUC 0.699, 95% CI 0.601–0.788). Chest pain was the only meaningful predictor. c) Good discrimination for pneumonia (AUC 0.787, 95% CI 0.728–0.849). Age was the most influential predictor, followed by BMI that contributed minimally. d) Good discrimination for detecting "Any Abnormality" (AUC 0.790, 95% CI 0.742–0.836). Any abnormality is used to describe any complication including but not limited to pneumonia, pleural effusion and cardiomegaly. Age was the primary predictor of any radiographic abnormality, followed by BMI and cough that contributed minimally. All other variables had negligible predictive importance.

Prediction models developed using routinely collected clinical metadata demonstrated good discriminative performance (Figure 5). The cardiomegaly model achieved the highest performance (Figure 5a), with an area under the receiver operating characteristic curve (AUC) of 0.881 (95% CI 0.807-0.963). The pneumonia model (Figure 5c) also demonstrated good discrimination (AUC 0.787, 95% CI 0.728-0.849), while the pleural effusion (Figure 5b) model achieved moderate performance (AUC 0.699, 95% CI 0.601-0.788). A composite model (Figure 5d) predicting the presence of any radiographic abnormality achieved an AUC of 0.790 (95% CI 0.742-0.836). Across all models, age consistently ranked among the strongest predictors of radiographic disease.

Accessibility and utilization of chest X-ray services

**Figure 6:**
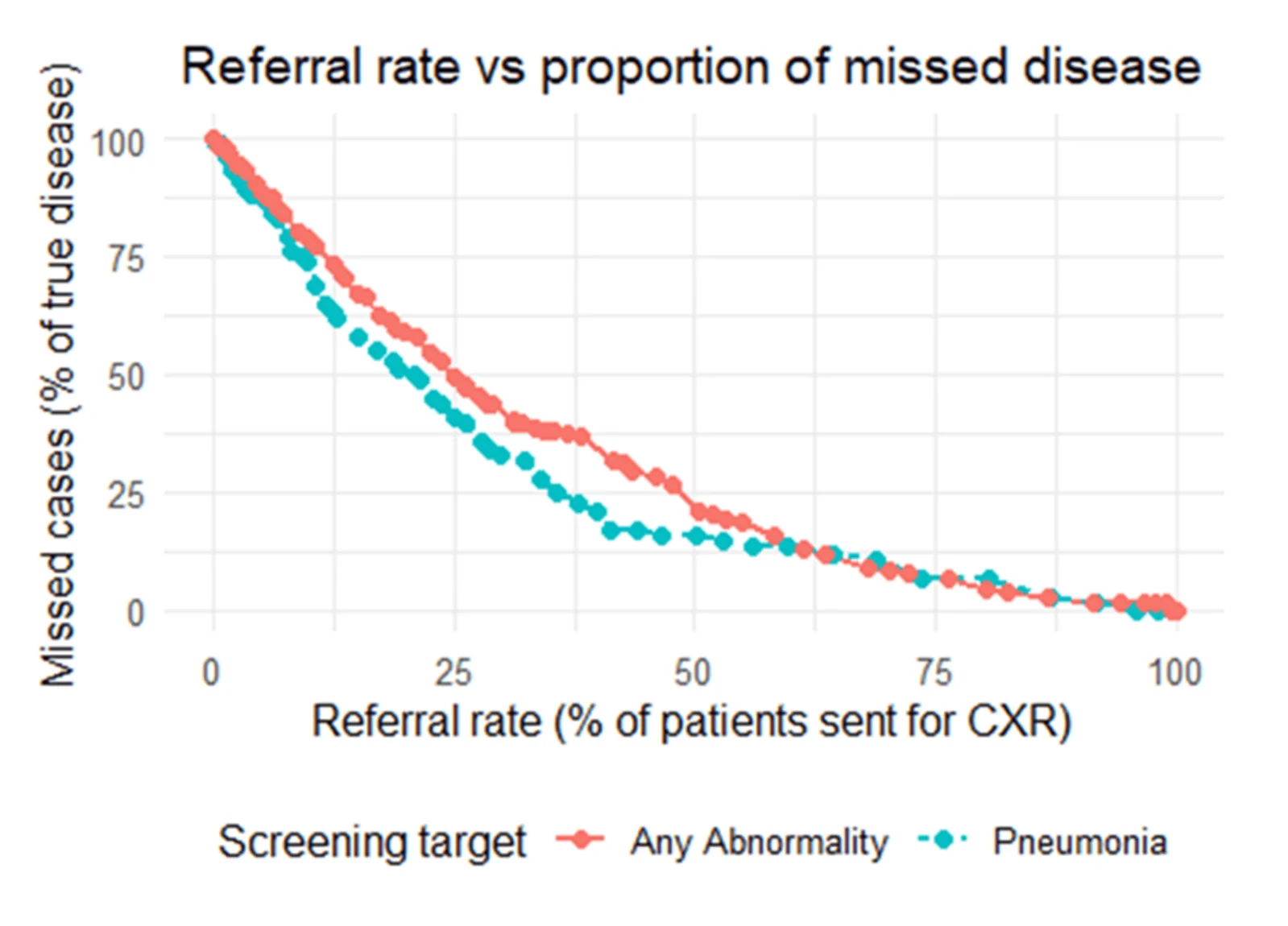
Referral rate and implication on missed diagnosis Diagnostic access cascade showing sequential structural barriers to chest X-ray utilization. The graph shows the screening target for pneumonia and any abnormality, which captures the presence of any clinically relevant radiographic abnormality warranting diagnostic attention, including but not limited to pneumonia, pleural effusion, cardiomegaly, and other actionable findings. CXR: Chest X-ray

Progressive reductions were observed across the diagnostic access pathway (Figure 6). Although 68% of participants reported that CXR services were available within their locality, this proportion decreased to 56% when personnel availability was considered and to 52% when affordability was taken into account. Ultimately, only 17% of participants indicated that they were both willing and able to undergo chest radiography despite perceived risks.

Facility-level comparisons demonstrated significant differences in referral pathways and personnel availability between Divine Mercy Hospital and Mbarara Regional Referral Hospital (Table 7). Walk-in attendance was more common at Divine Mercy Hospital, whereas willingness to undergo imaging was higher among participants attending Mbarara Regional Referral Hospital. Presentation at Mbarara Regional Referral Hospital was associated with referral status (adjusted OR 4.311, 95% CI 2.362-8.194; p <0.001), as was imaging-personnel availability (adjusted OR 3.343, 95% CI 1.511-7.813; p = 0.004), while travel time, affordability, and financing mechanisms were not significantly associated with referral patterns.

**Table 7:** Referral pathways and accessibility to the two hospitals

variable	p-value	Divine Mercy Hospital	Mbarara Regional Referral Hospital
Referral	2.45E-06	Walk-in (Direct) (92%)	Walk-in (Direct) (75%)
Financing	2.46E-01	Out of pocket (72%)	Out of pocket (81%)
X-ray nearby	6.46E-02	Yes (72%)	Yes (63%)
Personnel availability	2.20E-03	Yes (72%)	Yes (57%)
Affordability of X-ray	1.00E+00	Yes (74%)	Yes (75%)
Time to reach facility	3.99E-01	Less than 30 minutes (37%)	Less than 30 minutes (32%)
Still willing despite actors	2.69E-04	No (74%)	No (56%)

Exploratory stage 1 referral simulations

The study-sample simulations illustrated the trade-off between imaging demand and case detection at the selected operating thresholds. These results describe model behavior within the study dataset and do not represent prospectively evaluated clinical performance (Tables 8, 9).

**Table 8:** Stage 1 operating policies for pneumonia screening (prevalence = 18.9%)

Policy	Referral rate	Sensitivity	Missed cases	Specificity	Positive predictive value (PPV)	Negative predictive value (NPV)
	(%)	(%)	(%)	(%)	(%)	(%)
Capacity-constrained	35.7	75	25	73.4	39.6	92.7
Safety-prioritized	73.7	93.4	6.6	30.9	23.9	95.3

**Table 9:** Stage 1 operating policies for any abnormality screening (prevalence = 35.6%)

Policy	Referral rate	Sensitivity	Missed cases	Specificity	Positive predictive value (PPV)	Negative predictive value (NPV)
	(%)	(%)	(%)	(%)	(%)	(%)
Capacity-constrained	35.1	62	38	79.8	62.9	79.2
Safety-prioritized	67.9	90.8	9.2	44.7	47.6	89.8

For pneumonia, a capacity-constrained policy referred approximately 36% of patients while identifying 75% of cases. A safety-prioritized strategy increased case detection to more than 93% but substantially increased referral rates. Similar patterns were observed for the detection of any radiographic abnormality, with higher sensitivity achieved at the expense of increased imaging utilization (Figure 6).

## Discussion

This study examined pulmonary disease patterns, associated risk profiles, exploratory prediction-model discrimination, and barriers to diagnostic imaging using a multimodal CXR dataset from patients receiving routine clinical care in southwestern Uganda. By integrating radiographic findings with structured clinical metadata and health-system access variables, the study extends beyond traditional disease surveillance to provide preliminary insights into diagnostic decision-making in a resource-limited setting. The findings suggest that routinely obtainable clinical information may contribute to disease characterization, preliminary risk stratification, and the exploration of strategies for prioritizing imaging services. The study also highlights structural barriers affecting access to chest radiography. These observations are consistent with the increasing recognition that multimodal datasets are important for developing clinically relevant and context-appropriate diagnostic-support systems, particularly in LMICs [14].

Pneumonia was the most frequently reported radiographic abnormality, followed by pleural effusion and cardiomegaly. This distribution reflects the continuing burden of infectious respiratory disease in Uganda and the growing coexistence of chronic cardiothoracic conditions [15]. Similar epidemiological transitions have been observed in many LMICs, where infectious diseases remain prevalent alongside a rising burden of noncommunicable diseases. Consequently, healthcare systems are increasingly required to diagnose and manage patients with multiple interacting disease processes rather than isolated conditions [16]. The predominance of pneumonia in this study further emphasizes the importance of strengthening timely diagnostic and referral pathways for lower respiratory tract infections, particularly in settings with limited access to imaging services.

A notable observation was the considerable overlap in presenting symptoms across the major radiographic abnormalities. Cough, chest pain, difficulty breathing, fever, and weight loss were frequently reported across different radiographic outcomes, demonstrating the limited specificity of symptom-based assessment alone. These findings are consistent with previous studies showing that pulmonary diseases often present with shared clinical manifestations [17], making differentiation based solely on symptoms difficult without radiographic confirmation. The frequent coexistence of multiple radiographic abnormalities further illustrates the complexity of cardiothoracic disease in routine clinical practice and the limitations of relying on individual clinical features to guide diagnostic decisions. Collectively, these findings support the integration of clinical assessment with imaging and other diagnostic information to strengthen diagnostic decision-making and potentially reduce missed or delayed diagnoses [18].

Age was the most consistent predictor of pneumonia and cardiomegaly in the multivariable analyses, although it was not independently associated with pleural effusion. This finding is biologically plausible and aligns with previous epidemiological studies demonstrating increased susceptibility to respiratory infections and cardiovascular disease with advancing age. Progressive immunosenescence, cumulative environmental exposures, declining physiological reserve, and increasing multimorbidity may contribute to elevated disease risk among older adults [19,20]. Body mass index was also associated with certain radiographic outcomes, although the direction of association differed between diseases. Higher body mass index was associated with slightly increased odds of cardiomegaly and reduced odds of pleural effusion. These findings suggest that nutritional status and body composition may influence different pathological processes through distinct mechanisms. However, because some effect estimates were close to the null and the number of outcome events was limited, these associations should be interpreted cautiously.

Socioeconomic indicators such as education and income were not independently associated with the selected radiographic abnormalities after adjustment for other variables. Their effects may operate through more proximal determinants, including environmental exposure, healthcare utilization, underlying clinical status, and the ability to access diagnostic services, rather than through direct relationships with radiographic disease. However, the absence of statistically significant associations should not be interpreted as evidence that socioeconomic circumstances are unimportant. The study may have had limited statistical power to detect modest relationships, and the selected sample comprised patients who had already entered a chest-radiography referral pathway.

The exploratory prediction models based on clinical metadata showed apparent discrimination within the study sample, particularly for cardiomegaly and pneumonia. These results suggest that information available during routine patient assessment may contain signals associated with radiographer-reported abnormalities before imaging is performed. However, the reported AUCs were obtained from the model-development sample and were not corrected for optimism through internal validation, assessed for calibration, or evaluated in an external population. They may therefore overestimate performance in new patients, particularly for cardiomegaly, for which the number of outcome events was small. The findings should consequently be interpreted as preliminary and hypothesis-generating rather than evidence of a validated clinical prediction tool.

Similar studies have suggested that integrating clinical metadata with imaging information may improve diagnostic assessment and provide a more complete representation of the patient than approaches based on imaging alone [21]. In the present study, however, the clinical metadata models were intended only to explore pre-imaging risk stratification. Radiographer-reported findings were used as outcome labels and were not included as predictors in the referral models. The proposed approach is therefore not intended to replace chest radiography, radiographers, expert image interpretation, or clinical judgement. Radiographers remain essential for image acquisition and routine imaging-service delivery. If subsequently validated, a clinical metadata-based approach might complement existing workflows by supporting the prioritization of patients for imaging in settings where equipment, personnel, and financial resources are limited.

Beyond disease characterization, the study provides insights into barriers affecting access to CXR services. Although imaging services were geographically available to many participants, effective access declined after personnel availability, affordability, and willingness to undergo imaging were considered. Only a relatively small proportion of participants remained both willing and able to complete the diagnostic pathway. These findings indicate that diagnostic access is influenced by multiple interconnected health-system factors rather than by the availability of equipment alone. Similar challenges have been reported across sub-Saharan Africa, where shortages of trained personnel, inadequate financing mechanisms, and inequitable distribution of diagnostic services continue to impede timely access to medical imaging [3]. Addressing these barriers will require coordinated investment in workforce development, financing mechanisms, infrastructure, referral systems, and community engagement.

The study also explored a proposed two-stage diagnostic framework for resource-limited settings. In the first stage, routinely obtainable clinical metadata would be used to estimate the probability of a radiographic abnormality and support decisions about prioritization for chest radiography. In a possible future second stage, image-based AI models could assist with interpretation of acquired images. The present study evaluated only the exploratory clinical metadata component; it did not develop or evaluate an image-based AI model. The image-based component should therefore be understood as a proposed direction for future work rather than as a component validated in this study.

The Stage 1 referral simulations illustrated the trade-off between imaging demand and case detection under selected operating thresholds. Within the study sample, the capacity-constrained and safety-prioritized policies produced different referral volumes and proportions of missed cases. These simulations demonstrate how changing a decision threshold may alter the balance between conserving imaging capacity and identifying patients with recorded abnormalities. Nevertheless, the operating thresholds were selected as post hoc illustrative scenarios and were not prospectively implemented or clinically validated. The results therefore do not demonstrate that metadata-based triage safely reduces imaging demand, improves access, optimizes resource allocation, or improves patient outcomes. Rather, they provide a preliminary framework for developing and prospectively evaluating alternative referral strategies. Comparable multimodal workflows that combine structured clinical information with imaging have been proposed as potential approaches for integrating AI into clinical practice [18], but their safety, effectiveness, and operational value must be established in the intended healthcare setting.

The strengths of this study include the integration of radiographic findings with demographic, clinical, behavioral, environmental, and health-system information collected under routine clinical conditions. Unlike many publicly available CXR datasets that focus primarily on image classification, this dataset captures the broader clinical and health-system context in which imaging decisions are made. It therefore provides opportunities to investigate disease patterns, associated clinical factors, healthcare utilization, barriers to diagnostic access, and potential referral pathways. The inclusion of participants from two levels of healthcare delivery also provides useful preliminary information about how diagnostic access and referral processes operate across different facilities in southwestern Uganda.

Several limitations should be considered when interpreting the findings. First, the study used a relatively modest sample from two healthcare facilities, with radiographs obtained primarily at one facility. This limits geographical and institutional generalizability. Furthermore, participants had already been referred for chest radiography because of suspected pulmonary or cardiothoracic disease. This may have introduced referral-selection and spectrum bias and limits applicability to community-screening settings, primary-care populations, and patients not already selected for imaging.

Second, the radiographic outcomes were derived from routine reports prepared by radiographers rather than from independent consensus interpretation by expert radiologists. The rule-based procedure used to convert narrative reports into binary outcome labels was also not formally validated against manual classification or an independent reference standard. Potential inter-reader variability and outcome-label misclassification may therefore have influenced the estimated disease frequencies, multivariable associations, and apparent model discrimination.

Third, missing covariate data were handled using complete-case analysis without imputation, resulting in differences in analytical sample sizes across the outcome-specific models. If missingness was associated with participant characteristics or disease status, complete-case analysis may have introduced bias. The relatively small number of outcome events, particularly for cardiomegaly, also increased the risk of model overfitting, coefficient instability, and optimistic estimates of discrimination. In addition, the exploratory regression analyses did not include comprehensive assessment of all potential nonlinear relationships, interactions, multicollinearity, or influential observations.

Fourth, the reported AUCs represent apparent discrimination within the model-development sample. The models were not corrected for optimism through internal validation, assessed for calibration, evaluated using a held-out test set, or validated in an external population. The results therefore do not establish model calibration, transportability, clinical usefulness, or expected performance in new patients. The absence of image-derived features is an additional limitation, although the clinical metadata models were intended to examine pre-imaging risk stratification rather than image interpretation.

Finally, the referral policies were exploratory, post hoc simulations rather than prospectively implemented clinical interventions. They were not evaluated for clinical safety, workflow feasibility, cost-effectiveness, effects on equity, or patient outcomes. Consequently, the simulations illustrate potential trade-offs between referral volume and case detection but do not establish the effectiveness of the proposed referral framework in routine practice.

Future research should expand recruitment to additional healthcare facilities and more geographically diverse populations, increase the number of outcome events, establish standardized image annotation using independent expert-radiologist review, and formally validate the rule-based report-classification procedure. Prediction models should undergo appropriate internal validation, optimism correction, calibration assessment, and external validation before clinical application. Prospective implementation studies will also be necessary to determine whether clinical metadata-based prioritization can be integrated safely into existing workflows and whether it improves diagnostic access, efficiency, equity, or patient outcomes. Subsequent research may then evaluate a fully integrated multimodal system combining validated clinical metadata models with image-based AI tools.

Overall, this exploratory pilot study highlights the value of integrating clinical metadata, radiographer-reported findings, and health-system information to improve understanding of pulmonary disease patterns and diagnostic pathways in southwestern Uganda. Routinely obtainable clinical information may have potential to support preliminary risk stratification before imaging, while multimodal datasets provide a useful foundation for developing locally relevant diagnostic-support tools. However, the present findings do not establish a validated or clinically effective referral system. Independent verification of radiographic outcomes, formal validation of the report-classification procedure, internal and external model validation, calibration assessment, and prospective workflow evaluation are required before the proposed approach can support patient-level referral decisions in routine clinical practice.

## Conclusions

This exploratory pilot study examined radiographer-reported pulmonary and cardiothoracic abnormalities, associated clinical factors, and barriers to chest X-ray use among patients already referred for radiography in southwestern Uganda. Pneumonia emerged as the most frequently reported abnormality, with increasing age linked to both pneumonia and cardiomegaly. The significant overlap in clinical presentation across radiographic outcomes underscores the limited specificity of symptoms alone and emphasizes the importance of chest radiography in evaluating suspected cardiothoracic disease. Routinely obtainable clinical metadata demonstrated potential for preliminary pre-imaging risk stratification, while Stage 1 simulations indicated possible trade-offs between imaging-referral volume and case detection. However, the reported model performance reflects apparent discrimination within the study sample, and the simulated referral strategies were neither prospectively implemented nor clinically validated. Consequently, these findings are hypothesis-generating and do not establish a validated, safe, or clinically effective referral tool. Further research should encompass larger and more geographically diverse populations, independent expert-radiologist verification of radiographic outcomes, formal validation of the report-classification procedure, and appropriate internal and external validation of the prediction models, including calibration assessment. Prospective studies are also necessary to evaluate workflow feasibility, clinical safety, equity, and patient outcomes. Following such validation, integrating clinical metadata with image-based AI models may facilitate the development of context-appropriate diagnostic systems for resource-constrained healthcare settings.

## References

[1] Al-Qaness MA, Zhu J, Al-Alimi D (2024). Chest X-ray images for lung disease detection using deep learning techniques: a comprehensive survey. Arch Comput Methods Eng.

[2] Nakrani H, Shahra EQ, Basurra S (2025). Advanced diagnosis of cardiac and respiratory diseases from chest X-ray imagery using deep learning ensembles. J Sens Actuator Networks.

[3] Karera A, Davidson F, Engel-Hills P (2024). Operational challenges and collaborative solutions in radiology image interpretation: perspectives from imaging departments in a low-resource setting. J Med Radiat Sci.

[4] Ahmad HK, Milne MR, Buchlak QD (2023). Machine learning augmented interpretation of chest X-rays: a systematic review. Diagnostics (Basel).

[5] Brady AP (2017). Error and discrepancy in radiology: inevitable or avoidable?. Insights Imaging.

[6] Adams SJ, Henderson RD, Yi X, Babyn P (2021). Artificial intelligence solutions for analysis of X-ray images. Can Assoc Radiol J.

[7] Ade-Ibijola A, Okonkwo C (2023). Artificial intelligence in Africa: emerging challenges. Responsible AI in Africa: Challenges and Opportunities.

[8] Najjar R (2023). Redefining radiology: a review of artificial intelligence integration in medical imaging. Diagnostics (Basel).

[9] Musa A, Prasad R, Onwualu P (2026). A systematic review of cross-population shifts in medical imaging analysis with deep learning. Big Data Cogn Comput.

[10] Tripathi S, Gabriel K, Dheer S (2023). Understanding biases and disparities in radiology AI datasets: a review. J Am Coll Radiol.

[11] Willemink MJ, Koszek WA, Hardell C (2020). Preparing medical imaging data for machine learning. Radiology.

[12] Alabduljabbar A, Khan SU, Alsuhaibani A (2024). Medical imaging datasets, preparation, and availability for artificial intelligence in medical imaging. AI Data Rev.

[13] Tavaziva G, Harris M, Abidi SK (2022). Chest X-ray analysis with deep learning-based software as a triage test for pulmonary tuberculosis: an individual patient data meta-analysis of diagnostic accuracy. Clin Infect Dis.

[14] Boum Y 2nd, Atwine D, Orikiriza P, Assimwe J, Page AL, Mwanga-Amumpaire J, Bonnet M (2014). Male gender is independently associated with pulmonary tuberculosis among sputum and non-sputum producers people with presumptive tuberculosis in Southwestern Uganda. BMC Infect Dis.

[15] Mubuuke AG, Businge F, Kiguli-Malwadde E (2019). Diagnostic accuracy of chest radiograph interpretation by graduate radiographers in Uganda. Afr J Health Profession Educ.

[16] (2023). Global burden of chronic respiratory diseases and risk factors, 1990-2019: an update from the Global Burden of Disease Study 2019. EClinicalMedicine.

[17] Nalunjogi J, Mugabe F, Najjingo I (2021). Accuracy and incremental yield of the chest X-ray in screening for tuberculosis in Uganda: a cross-sectional study. Tuberc Res Treat.

[18] Yang L, Wan Y, Pan F (2025). Enhancing chest X-ray diagnosis with a multimodal deep learning network by integrating clinical history to refine attention. J Imaging Inform Med.

[19] Cilloniz C, Ceccato A, San Jose A, Torres A (2016). Clinical management of community acquired pneumonia in the elderly patient. Expert Rev Respir Med.

[20] Torres A, Cillóniz C, Blasi F (2018). Burden of pneumococcal community-acquired pneumonia in adults across Europe: a literature review. Respir Med.

[21] Grant D, Papież BW, Parsons G (2021). Deep learning classification of cardiomegaly using combined imaging and non-imaging ICU data. Medical Image Asymmetry and Machine Learning in Clinical Diagnostics.

